# Management of high-grade ovarian adenocarcinoma in an intraperitoneal pelvic renal transplant recipient^☆^

**DOI:** 10.1016/j.gore.2025.101726

**Published:** 2025-04-16

**Authors:** Hadi Erfani, Esra Demirel, Farr Nezhat

**Affiliations:** a University of Southern California/Los Angeles General Medical Center, Division of Gynecologic Oncology, Los Angeles, CA, USA; b NYU Langone-Long Island School of Medicine, Mineola, NY, USA; c Nezhat Surgery for Gynecology/ Oncology, NYU Langone-Long Island School of Medicine, and Weill Cornell Medical College, New York, NY, USA

**Keywords:** Ovarian cancer, Kidney transplant, Gynecologic oncology, Surgical management, Immunosuppression

## Abstract

**Background:**

The increasing number of organ transplant recipients has led to a rise in cancer diagnoses in this population. Kidney transplant recipients, in particular, have a two-to-four-fold higher risk of developing cancer due to immunosuppressive therapy. The management of gynecologic cancers in this subset of patients presents unique challenges due to anatomical considerations, immunosuppression, and the nephrotoxicity of oncologic treatments.

Case Presentation.

A 44-year-old woman with a history of polycystic kidney disease underwent a bilateral nephrectomy followed by a living-related intraperitoneal renal transplant. She presented with pelvic pain and was found to have high-grade ovarian adenocarcinoma, possibly arising from endometriosis. Surgical staging included total laparoscopic hysterectomy, bilateral salpingo-oophorectomy, omentectomy, and right pelvic lymph node dissection. The patient underwent six cycles of carboplatin and paclitaxel chemotherapy while maintaining stable renal function with close monitoring of immunosuppressive drug levels. Surveillance imaging detected recurrence in the right pelvic lymph nodes, leading to secondary tumor debulking and whole pelvic radiation therapy. Despite a slight increase in creatinine, kidney function remained stable, and the patient has remained disease-free on subsequent follow-ups.

**Conclusion:**

The management of ovarian cancer in kidney transplant recipients requires a multidisciplinary approach to balance oncologic control with graft preservation. Close monitoring of renal function, careful surgical planning, and tailored chemotherapy and radiation strategies are critical. More research is needed to establish standardized guidelines for managing gynecologic malignancies in transplant patients.

## Highlights

• Immunosuppressive therapy complicates cancer management in transplant patients.
• Surgical staging and adjuvant chemotherapy were successfully performed while maintaining renal graft function.
• Recurrence was managed with secondary tumor debulking and pelvic radiation.
• A multidisciplinary approach is essential for optimizing both oncologic and transplant outcomes.
• Further research is necessary to guide management strategies for gynecologic cancers in transplant patients.

Dear Editor,

I am pleased to submit our video submission titled **“Management of High-Grade Ovarian Adenocarcinoma in an Intraperitoneal Pelvic Renal Transplant Recipient”** for consideration for publication in *Gynecologic Oncology Reports.* This submission highlights the unique challenges in managing gynecologic malignancies in kidney transplant recipients, emphasizing surgical techniques, oncologic considerations, and renal graft preservation strategies.

Given the increasing number of solid organ transplant recipients, the intersection of gynecologic oncology and transplant medicine is becoming increasingly relevant. We believe this video will serve as a valuable educational resource for gynecologic oncologists, transplant surgeons, and multidisciplinary teams facing similar clinical scenarios.

All authors have contributed significantly to the content, and there are no conflicts of interest to disclose. We appreciate your time and consideration and look forward to the opportunity to contribute to the ongoing discussion in gynecologic oncology.

Thank you for your consideration. Please do not hesitate to reach out if any additional information is needed.

Sincerely,

Farr Nezhat, MD.

Nezhat Surgery for Gynecology/ Oncology.

Weill Cornell Medical College.

NYU Langone-Long Island School of Medicine.

## Funding Statement

This research received no specific grant from any funding agency in the public, commercial, or not-for-profit sectors.

## CRediT authorship contribution statement

**Hadi Erfani:** Conceptualization, Investigation, Methodology, Writing – original draft, Writing – review & editing. **Esra Demirel:** Writing – review & editing. **Farr Nezhat:** Conceptualization, Supervision, Writing – review & editing.

